# Case report: anti-glomerular basement membrane antibody disease with normal renal function

**DOI:** 10.1186/s12882-015-0179-1

**Published:** 2015-11-04

**Authors:** China Nagano, Yoshimitu Goto, Katuaki Kasahara, Yoshiyuki Kuroyanagi

**Affiliations:** Japanese Red Cross Nagoya Daini Hospital, 2-9 Myoken-cho, Showa-ku, Nagoya City, Aichi Prefecture 466-8650 Japan

**Keywords:** Anti-GBM, Pediatrics, Normal renal function, School urine screening program, case report

## Abstract

**Background:**

Anti-glomerular basement membrane (GBM) antibody disease is a rare autoimmune disorder characterized by rapidly progressive glomerulonephritis caused by autoantibodies against the α3-chain of type IV collagen in the GBM.

**Case presentation:**

An 8-year-old girl with hematuria and proteinuria due to anti-GBM nephritis was diagnosed with hematuria and proteinuria during a school urine screening program. Her blood pressure and serum creatinine levels were normal. Her hematuria and proteinuria persisted for several months. Since a spot urine protein to creatinine ratio was around 7 g/g Cre, a percutaneous renal biopsy was performed. Immnofluorescent staining demonstrated a linear pattern for immunoglobulin G along the entire GBM. Chest computed tomography was normal. Anti-GBM antibody assays were reported as slightly raised; thus, the diagnosis was anti-GBM disease with normal renal function. Treatment included plasma exchange, intravenous high-dose methylprednisolone, and cyclophosphamide as a mainstay medication. The treatment was rapidly effective with an immediate decrease in anti-GBM titers and proteinuria.

**Conclusions:**

Cases of anti-GBM disease with normal renal function in children are rare. Treatment in children has not been established; therefore, clinicians need to carefully select an effective treatment because the prognosis is poor.

## Background

Anti-glomerular basement membrane (GBM) disease is included among immune complex small vessel vasculitides. This disease is a vasculitis that affects the glomerular capillaries, pulmonary capillaries, or both, with GBM deposition of anti-GBM autoantibodies. Lung involvement causes pulmonary hemorrhage, and renal involvement causes glomerulonephritis with necrosis and crescents [1]. It has a frequency of 0.5–1 case per million/year. All age groups can be affected, but the peak incidence occurs in the third decade in young men with a second peak in the sixth and seventh decades, which affects men and women equally. Although anti-GBM disease in childhood is quite uncommon, we observed anti-GBM disease in an 8-year-old girl with normal renal function who had participated in a school urine screening program.

## Case presentation

**Fig. 1:**
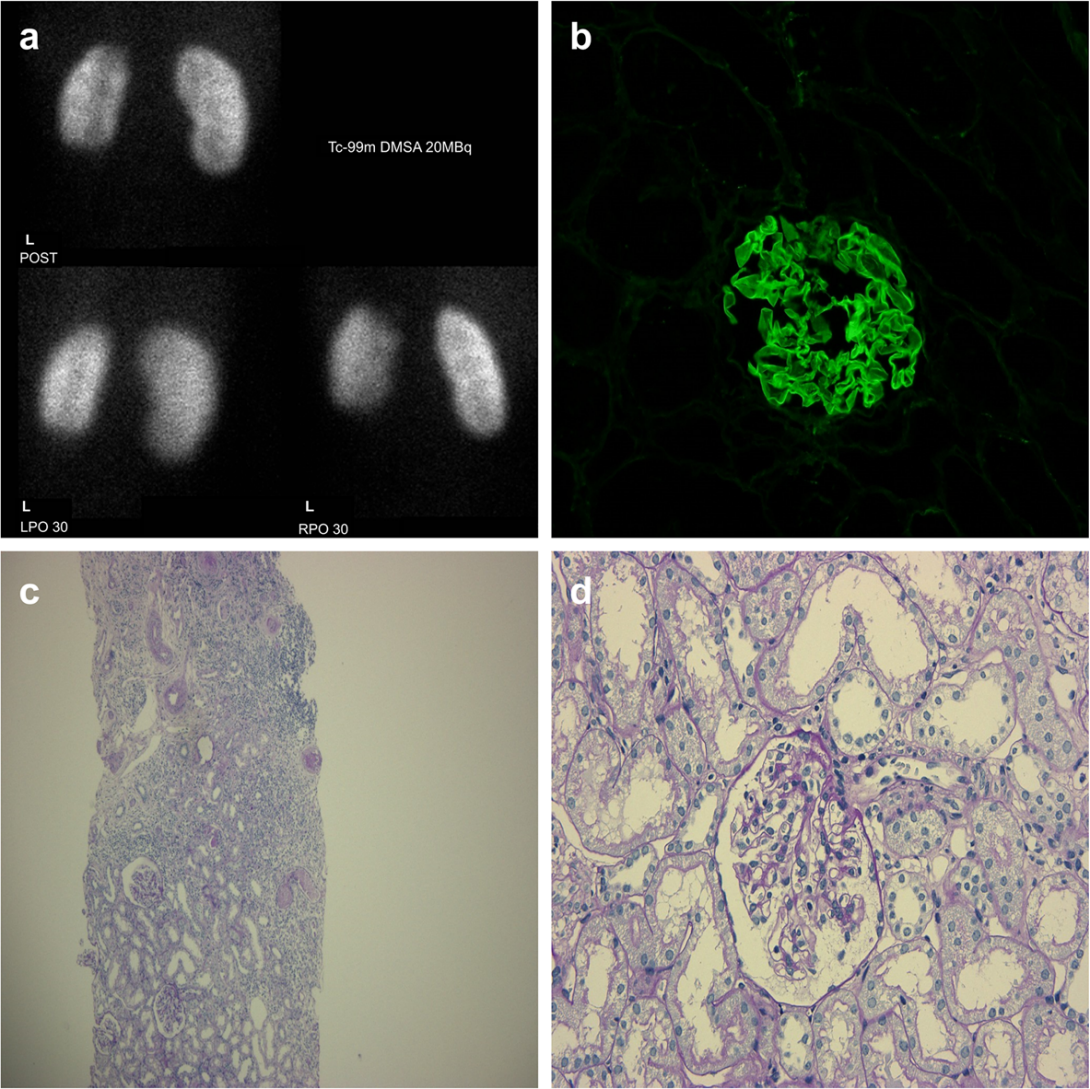
**a** Dimercaptosuccinic acid scan. **b** Immunofluorescence showing linear staining of immunoglobulin G along the glomerular basement membrane. **c** Periodic acid-Schiff stain showing a scar (×40). **d** Periodic acid-Schiff stain showing no crescent formation (×200)

An 8-year-old girl was diagnosed with hematuria and proteinuria during a school urine screening program. She was referred to the medical outpatient clinic. Her clinical examination and serum creatinine level were normal. She had no medical or family history of this condition. A spot urine protein to creatinine ratio was around 7 g/g Cre. She was admitted to our department because of persistent hematuria and proteinuria. Again, the clinical examination was normal. Results of the blood tests were as follows: white blood cell count, 10.6 × 10^9^/L; hemoglobin level, 11.6 g/dL; platelet count, 240 × 10^9^/L; sodium level, 135 mmol/L; potassium level, 3.7 mmol/L; total protein level, 5.56 g/dL; albumin level, 2.67 g/dL; urea, 13.9 mg/dL; creatinine level, 0.40 mg/dL; triglyceride level, 56 mg/dL; total cholesterol level, 267 mg/dL; complement component (C) 3 level, 104 mg/dL; C4, 29 mg/dL; total complement (CH50) level, 36.6 U/mL; immunoglobulin (Ig)-G level, 766 mg/dL; and IgA level 137 mg/dL. At admission, urinalysis showed microscopic hematuria and proteinuria. A spot urine protein to creatinine ratio was 8.6 g/g Cre. During a routine inspection using dimercaptosuccinic acid scan (DMSA) before renal biopsy, Tc-99 m DMSA images showed a focal decreased uptake in the upper and lower portion of the left kidney (Fig. 1a).

A renal biopsy was performed from the left kidney. On light microscopy, the patient had 12 glomeruli with no crescent formation. The glomeruli showed mild proliferation of the mesangial cells. Immunofluorescence showed linear staining of IgG along the GBM (Fig. 1b–d). Thus, anti-GBM antibody glomerulonephritis and Goodpasture syndrome were suspected, and the serological workup (enzyme-linked immunosorbent assay) was positive for anti-GBM antibody elevation with a value of 29.6 U/mL (normal, <2 U/mL). Anti-nuclear and anti-double-stranded deoxyribonucleic acid antibodies, antineutrophil antibody, antineutrophil cytoplasmic antibody, anti-proteinase 3, hepatitis B, and hepatitis C serologies were negative. The complement levels were normal. Renal ultrasonography was normal. Chest computed tomography did not show diffuse alveolar hemorrhage. All the labs and pathology suggested anti-GBM disease.

Treatment consisted of three sessions of double-filtration plasmapheresis (DFPP) every other day. Her anti-GBM antibody level decreased to <2.0 U/mL. Intravenous pulse methylprednisolone (30 mg/kg/day) was administered for three days, and prednisone (2 mg/kg every day on a weaning regimen) was continued. In addition, oral cyclophosphamide (2 mg/kg/day daily for 8 weeks) was administered. The treatment was rapidly effective with an immediate decrease in anti-GBM titers and proteinuria. There were no side effects from the treatment. There was a scar on the left kidney, which was observed using DMSA. Her bladder pressure was measured after 6 months of treatment, and we found low compliance in the bladder, vesicoureteral reflux (VUR)-2 of the right kidney, and VUR3 of the left kidney.

## Conclusions

This case of anti-GBM disease demonstrated the following three points. First, she had anti-GBM disease with normal kidney function. Second, VUR with bladder dysfunction may have been the cause. Third, DFPP is an effective treatment for anti-GBM disease with normal kidney function.

Anti-GBM disease remains a very uncommon condition in the pediatric population. We identified only 23 cases published in English literature over a 25-year span [2]. Anti-GBM disease has an estimated frequency of 0.5–1 case per million inhabitants/year, according to a series published in New Zealand, Australia, the United Kingdom, the United States, China, and Scandinavia [3, 4]. We described the case of an 8-year-old girl with only minimal renal involvement and no pulmonary symptoms.

Symptoms of nephritis often exhibit a clinical picture of rapidly progressive glomerulonephritis; however, to date, several cases of anti-GBM disease with normal kidney function have been reported. The prevalence is approximately 3–36 % [5]. Systemic symptoms (i.e., malaise, fever, or weight loss) were less common in patients with normal renal function than in those with renal impairment. Circulating anti-GBM antibodies were detected less often and at lower levels in individuals with normal renal function than in those with renal impairment. The histological abnormalities were not as marked in patients with normal renal function compared to those with renal impairment [6]. The IgG subclasses for the distribution of natural autoantibodies were restricted to IgG2 (100 %) and IgG4 (100 %), whereas in patients, it was mainly IgG1 (93.8 %) and IgG4 (90.6 %) [7]. Different IgG subclasses have different biological properties. For example, IgG4, unlike IgG1, does not efficiently bind to C1q; therefore, it does not activate the classical pathway of the complement. In addition, the IgG Fc receptors on mononuclear macrophage poorly bind to IgG4. Thus, IgG4 is unlikely to trigger severe inflammatory damage to the glomeruli. Previously, we observed anti-GBM antibodies in patients with mild renal dysfunction (serum creatinine level, <300 mmol/L) that were predominantly of the IgG4 subclasses (75 %), which supports the speculation that IgG subclasses of anti-GBM antibodies may be associated with different clinical presentations and disease progression [5]. Additionally, since a school urine screening system is widespread in Japan, it was possible to discover this condition early in our case.

Direct damage to the kidneys due to reflux caused anti-GBM antibody production.

In adults, a wide variety of agents have been linked to anti-GBM disease, including automobile exhaust, fuels (gasoline and jet fuel), paints, solvents (organic, degreasing, dry-cleaning, and paint solvents), hair products (hair spray and hairdressing solvents), cleaners, glue, and insecticides [8]. There have been case reports on anti-GBM disease following lithotripsy and ureteric obstruction, suggesting that antigens released from a mechanically damaged kidney may initiate the disease in susceptible individuals [9]. There may be a case in which antibody production is caused by reflux nephropathy in VUR.

We used DFPP as therapy, and the antibodies became negative quickly. Recommendations for the treatment of anti-GBM disease are mainly based on studies in adults. The treatment of choice in anti-GBM disease is plasma exchange combined with prednisone and cyclophosphamide. Plasma exchange can remove the anti-GBM antibodies along with other inflammation mediators while the immunomodulators lower antibody formation. We chose DFPP because the antibody titer is low, and we selected DFPP since it is efficient in terms of infection and cost. In previous reports, DFPP was effective for treating anti-GBM disease [10, 11]. In fact, in our case, IgG removal efficiency was 60 % after the first DFPP procedure, and the antibody was negative after DFPP was performed three times. If the antibody titer is high, plasmapheresis is superior to DFPP, and it is considered an effective treatment that can be performed safely even if the antibody titer is low. When the antibody titer is high, simple plasma exchange is superior to DFPP. However, when the antibody titer is low, DFPP is an effective treatment because it is safer and less expensive.

In a previous nationwide survey in Japan, the prognosis was 80 % for anti-GBM type nephritis and 60 % for Goodpasture syndrome, and the renal prognosis was 50 % for anti-GBM type nephritis and 40 % for Goodpasture syndrome. Prognosis seems to worsen if the patient is oliguric at presentation, if the serum creatinine is >600 μmol/L (6.8 mg/dL), or if there is >50 % crescent formation of the glomeruli [2]. Disease recurrence with antibody production has been reported, but it is quite rare.

We reported on a pediatric case of anti-GBM disease with normal renal function. Because of the school urine screening program, it was discovered at an early stage. Direct damage to the kidney due to VUR is considered the etiology in the current case. It is interesting that in a case with renal failure due to reflux nephropathy with VUR, anti-GBM antibody is produced. We think that the accumulation of similar cases will help elucidate the pathology of anti-GBM disease in children.

## Consent

Written informed consent was obtained from the patient for publication of this case report. A copy of the written consent is available for review by the editor of this journal.

## Abbreviations

DFPP: double-filtration plasmapheresis
DMSA: dimercaptosuccinic acid scan
GBM: anti-glomerular basement membrane
Ig: immunoglobulin
PV: plasma volume
VUR: vesicoureteral reflux

## References

[1] Hellmark T, Segelmark M (2014). Diagnosis and classification of Goodpasture's disease (anti-GBM). J Autoimmun.

[2] Bayat A, Kamperis K, Herlin T (2012). Characteristics and outcome of Goodpasture's disease in children. Clin Rheumatol.

[3] Silvariño R, Noboa O, Cervera R (2014). Anti-glomerular basement membrane antibodies. Isr Med Assoc J.

[4] Salama AD, Levy JB, Lightstone L, Pusey CD (2001). Goodpasture's disease. Lancet.

[5] Cui Z, Zhao MH, Singh AK, Wang HY (2007). Antiglomerular basement membrane disease with normal renal function. Kidney Int.

[6] Ang C, Savige J, Dawborn J, Miach P, Heale W, Clarke B (1998). Anti-glomerular basement membrane (GBM)-antibody-mediated disease with normal renal function. Nephrol Dial Transplant.

[7] Cui Z, Wang HY, Zhao MH (2006). Natural autoantibodies against glomerular basement membrane exist in normal human sera. Kidney Int.

[8] Williamson SR, Phillips CL, Andreoli SP, Nailescu C (2011). A 25-year experience with pediatric anti-glomerular basement membrane disease. Pediatr Nephrol.

[9] Kluth DC, Rees AJ (1999). Anti-glomerular basement membrane disease. J Am Soc Nephrol.

[10] Kumazaki S, Umeda Y, Sato K, Mishima H, Ishihara T, Uzawa T. [Double filtration plasmapheresis in case of Goodpasture's syndrome]. Nihon Kyobu Shikkan Gakkai Zasshi. 1990;28:628–633. [Article in Japanese] 2214405

[11] Hajime N, Michiko A, Atsunori K, Tatsuo K, Yuko N, Naoki O (2009). A case report of efficiency of double filtration plasmapheresis in treatment of Goodpasture's syndrome. Ther Apher Dial.

